# Phylogenetic and characterization of the complete mitochondrial genome relationship of Argali sheep (*Ovis ammon*)

**DOI:** 10.1080/23802359.2019.1698369

**Published:** 2019-12-13

**Authors:** Cong Wang, Huailiang Xu, Diyan Li, Jiayun Wu, Anxiang Wen, Meng Xie, Qin Wang, Guangxiang Zhu, Qingyong Ni, Mingwang Zhang, Yongfang Yao

**Affiliations:** a College of Life Science, Sichuan Agricultural University, Ya’an, Sichuan, P. R. China;; b College of Animal Science and Technology, Sichuan Agricultural University, Chengdu, Sichuan, P. R. China

**Keywords:** Mitochondrial genome, Argali sheep, phylogenetic analysis

## Abstract

In this study, we sequenced the complete mitochondrial genome of Argali sheep (*Ovis ammon*). The total length was 16,612 bp, which contained 13 protein-coding genes (PCGs), 22 tRNA genes, 2 rRNA genes, and a control region (D-loop). Eight tRNA genes with one PCG (*ND6*) encoded on the L-strand, others were encoded on the H-strand. The neighbor-joining analysis shows that Argali sheep has a close relationship with the same genus species of *Ovis aries* and *Ovis orientalis*. This study has provided new data for the phylogeny of Argali sheep.

*Ovis ammon* (Ovis, Caprinae) also known as Argali sheep, is one of the members of Caprinae (Kumar et al. 2016; Smith and Xie 2010). In China, this gregarious animal is extensively distributed in the western mountain, including Junggar mountains, Tian Shan mountains, Pamirs, Altun mountains, Kunlun mountains, Qilian mountains, Qinghai-Tibet plateau, and Inner Mongolia plateau (Yu et al. 2008). The wild populations of Argali sheep are declining, so it was classified as near threatened species by International Union for Conservation of Nature (The IUCN Red List of Threatened Species 2008). Hence, our study reported its entire mitochondrial genome data, which had supplemented the mitochondrial genome data of the subfamily Caprinae.

The samples were collected from Xinjiang Uygur Autonomous Region (75°E ∼ 95°E; 35°N ∼ 50°N) and stored in Zoology laboratory of Sichuan Agricultural University (Accession:000745). The total genomic DNA was extracted from muscles using phenol-chloroform extraction method (Sambrook et al. 1990). The mitochondrial genome of Argali sheep was amplified and sequenced by 22 pairs of primers designed based on the conservative regions of *O. ammon* (HM236188). The sequenced data were edited and assembled by DNAstar (Swayne et al. 2015) and the phylogenetic tree was constructed using MEGA7.0 (Kumar et al. 2016).

The complete mitochondrial genome of Argali sheep is 16,612 bp (Genebank: MN564883), including 13 protein-coding genes (PCGs), 22 tRNA genes, 2 rRNA genes, and a control region (D-loop), which is similar to other species of Caprinae (Meadows et al. 2011). The base composition of mtDNA is A (33.67%), G (13.08%), T (27.29%), C (25.96%), so the percentage of A + T (60.96%) is higher than G + C (39.04%), A-T bias is more significant. Among the 13 PCGs, the longest one is 1821 bp (*ND5*) and the shortest is 201 bp (*ATP8*). Three PCGs (*ND2*, *ND3*, *ND5*) used ATA as start codon and the remainders were ATG. Six PCGs terminated with TAA, while *Cytb* terminated with AGA. The incomplete stop codon(T−) was observed in *ND1*, *ND2*, *ATP6*, *COX3*, *ND3*, and *ND4*. There were eight tRNA genes (*tRNA^Gln^*, *tRNA^Ala^*, *tRNA^Asn^*, *tRNA^Cys^*, *tRNA^Tyr^*, *tRNA^Ser^*, *tRNA^Glu^*, and *tRNA^Pro^*) with one PCG (*ND6*) encoded on the L-strand, and the other PCGs were encoded on the H-strand. The *12s rRNA* and *16s rRNA* were located between *tRNA^phe^* and *tRNA^leu^*, which was separated by *tRNA^val^*. In the sequence, we find one control region(CR), that is located between *tRNA^Pro^* and *tRNA^Phe^*.

The phylogenetic tree was constructed with the other 12 species from Caprinae, using *Bos taurus* as an outgroup (Figure 1). The phylogenetic analysis shows that Argali sheep has a close relationship with *Ovis aries* and *Ovis orientalis*, which belong to genus *Ovis*, and the topology structure was consistent with the previous study (Sun et al. 2017). This study provides new data for the phylogeny of Argali sheep.

**Figure 1.**
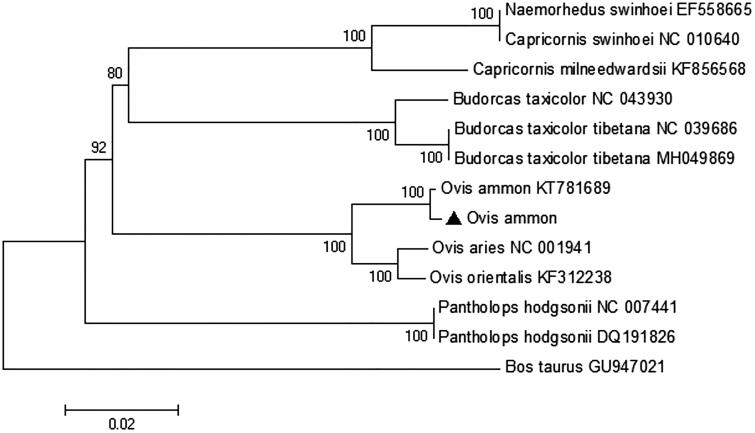
Neibour-joining(NJ) phylogenetic tree constructed based on 13 protein coding genes of *Ovis ammon* and other 12 Caprinae species, using *Bos taurus* as an outgroup and numbers at the branches indicated the bootstrapping values with 1000 replications. Solid triangle represents a sequence in this study.
